# Bacteriophage therapy for drug-resistant *Staphylococcus aureus* infections

**DOI:** 10.3389/fcimb.2024.1336821

**Published:** 2024-01-31

**Authors:** Kaixin Liu, Chao Wang, Xudong Zhou, Xudong Guo, Yi Yang, Wanying Liu, Rongtao Zhao, Hongbin Song

**Affiliations:** ^1^ College of Public Health, Zhengzhou University, Zhengzhou, China; ^2^ Chinese PLA Center for Disease Control and Prevention, Beijing, China; ^3^ College of Public Health, China Medical University, Shenyang, China

**Keywords:** bacteriophages, drug-resistant *Staphylococcus aureus*, infection, bacteriophage therapy, antimicrobial resistance

## Abstract

Drug-resistant *Staphylococcus aureus* stands as a prominent pathogen in nosocomial and community-acquired infections, capable of inciting various infections at different sites in patients. This includes *Staphylococcus aureus* bacteremia (SaB), which exhibits a severe infection frequently associated with significant mortality rate of approximately 25%. In the absence of better alternative therapies, antibiotics is still the main approach for treating infections. However, excessive use of antibiotics has, in turn, led to an increase in antimicrobial resistance. Hence, it is imperative that new strategies are developed to control drug-resistant *S. aureus* infections. Bacteriophages are viruses with the ability to infect bacteria. Bacteriophages, were used to treat bacterial infections before the advent of antibiotics, but were subsequently replaced by antibiotics due to limited theoretical understanding and inefficient preparation processes at the time. Recently, phages have attracted the attention of many researchers again because of the serious problem of antibiotic resistance. This article provides a comprehensive overview of phage biology, animal models, diverse clinical case treatments, and clinical trials in the context of drug-resistant *S. aureus* phage therapy. It also assesses the strengths and limitations of phage therapy and outlines the future prospects and research directions. This review is expected to offer valuable insights for researchers engaged in phage-based treatments for drug-resistant *S. aureus* infections.

## 1 Introduction

For nearly a century, antibiotics have played a pivotal role in humanity’s triumph over various infectious ailments. However, the unwarranted and improper utilization of antibiotics has spurred the rapid proliferation and dissemination of bacterial resistance, especially among multidrug-resistant (MDR) and extensively drug-resistant (XDR) bacteria. These strains present a substantial challenge to clinical infection management. A recent study, published in Lancet, has confirmed that in the year 2019, there were approximately 1.27 million global fatalities linked to bacterial antimicrobial resistance (AMR) (Murray et al., 2022). Furthermore, an estimated 4.95 million deaths were associated with bacterial AMR (Murray et al., 2022). Specifically, it was reported that 569,000 deaths (with a 95% UI of 406,000-771,000) were linked to bacterial AMR, and an additional 141,000 deaths (ranging from 99,900 to 196,000) were attributable to bacterial AMR within the 35 countries situated in the WHO Region of the Americas during the same year (Aguilar et al., 2023). It is worth noting that mortality related to antibiotic resistance has now escalated to become the third most prominent cause of death on a global scale (Murray et al., 2022). Furthermore, it is crucial to acknowledge that the incidence of AMR has notably exacerbated during the COVID-19 pandemic (Centers for Disease Control and Prevention, National Center for Emerging and Zoonotic Infectious Diseases, and Division of Healthcare Quality Promotion, 2022). Unfortunately, the development of novel antimicrobial agents lags significantly behind the emergence of drug-resistant bacteria (Butler Mark et al., 2022; Murray et al., 2022). The World Health Organization (WHO) has cautioned that, with the ongoing evolution of antibiotic resistance, the number of human fatalities attributable to multidrug-resistant bacterial infections is projected to surpass 10 million by 2050. This would exceed the mortality attributed to cancer, with an associated economic cost of $100 trillion (Miller and Liu, 2021; Blackman et al., 2022). In 2017, the WHO compiled a catalog of global priority pathogens necessitating exploration and development of innovative antimicrobial drugs (Karaman et al., 2020). Notably, Gram-positive (G+) bacteria constituted a significant proportion of the clinically resistant bacterial strains examined, particularly methicillin-resistant *Staphylococcus aureus* (MRSA), vancomycin-resistant *Enterococcus faecium* (VTEF), and β-lactamase-resistant *Streptococcus pneumoniae*, all of which pose considerable healthcare challenges (Karaman et al., 2020).

*Staphylococcus aureus* is a Gram-positive pathogenic bacterium and a key instigator of skin and soft tissue infections (Bitrus et al., 2018). These infections can culminate in tissue localization, purulent infections, and, if not effectively controlled, may lead to *Staphylococcus aureus* bacteremia (SaB) or even severe septic shock, with a SaB mortality rate as high as 25% (Wozniak et al., 2020). A recent study, as published in Lancet, has confirmed that *S. aureus* (emerged as the predominant bacterial factor contributing to mortality in 135 countries. Moreover, it was identified as the bacterium associated with the highest number of fatalities among individuals aged 15 and older on a worldwide scale (Ikuta et al., 2022). In the United States, *S. aureus* was found to affect an estimated 119,247 individuals, resulting in 19,832 deaths (Shahriar et al., 2019). In addition to the extensive secretion of virulence factors, biofilm formation is an important feature to protect *S. aureus* from host defense and eradication measures (Olsen, 2015; Paharik Alexandra and Horswill Alexander, 2016; Cangui-Panchi et al., 2022). Between 50 and 70% of nosocomial infections are caused by biofilm formation on implanted medical devices (Cangui-Panchi et al., 2022). *S. aureus* biofilm can protect cells from harsh conditions, including nutrient limitation, extreme temperature and dehydration, and even antibacterial drugs (Lee et al., 2020; Idrees et al., 2021; Guo et al., 2022; Cangui-Panchi et al., 2023). It is reported that bacterial cells covered by biofilm are 10-1000 times more resistant to antibiotics than their corresponding plankton forms (Paharik Alexandra and Horswill Alexander, 2016). Presently, clinical strategies for preventing and treating *S. aureus* infections predominantly hinge on antibiotic therapy. However, the imprudent use of antibiotics has resulted in a gradual increase in drug-resistant *S. aureus*, particularly in MRSA, which has a global presence and poses a substantial public health threat (Lázár et al., 2022). MRSA infections incur higher morbidity and mortality rates compared to methicillin-susceptible *Staphylococcus aureus* (MSSA) infections (Cascioferro et al., 2021), along with elevated treatment expenses and prolonged hospitalization periods. MRSA was responsible for over 100,000 deaths that can be attributed to AMR in the year 2019 (Murray et al., 2022). Presently, approximately 30% of hospital-acquired infections are attributed to MRSA, which displays resistance to a broad spectrum of antibiotics. Vancomycin has emerged as the most frequently employed drug and the final line of defense against MRSA infections (van Groesen et al., 2022). Nevertheless, with the rising incidence of MRSA infections and the extensive use of vancomycin, MRSA’s susceptibility to vancomycin has progressively waned, resulting in instances of vancomycin treatment failure (Tong et al., 2020). In a recent meta-regression model analyzing one-month mortality due to SaB, it was observed that MRSA exhibited a higher mortality rate when compared to non-resistant *S. aureus* (Bai et al., 2022). In addition, because MRSA can form biofilm on biological and abiotic surfaces, MRSA biofilm-associated infections are complex and difficult to eradicate (Idrees et al., 2021). The penetration of antibacterial drugs on biofilm is reduced, which makes MRSA survive in the presence of drugs at reduced concentrations (Idrees et al., 2021). Consequently, it is imperative to devise innovative infection-control strategies to combat drug-resistant *S. aureus* infections.

In recent years, bacteriophages (commonly known as “phages”) have garnered substantial attention as non-antibiotic agents with bactericidal properties in infection control (Jamal et al., 2019; Balcão et al., 2022; Weber-Dąbrowska et al., 2023). Phages are viruses capable of infecting and lysing bacteria (Li et al., 2023). They can rapidly replicate and proliferate following specific invasions of host bacteria, subsequently releasing endolysins (referred to as “lysins”) to disrupt bacterial cells, thereby achieving antibacterial effects. As early as 1919, a decade prior to the discovery of antibiotics, French scientist Félix d’Hérelle employed phages for the first time to treat dysentery and other infectious diseases, yielding positive outcomes (Chanishvili, 2012). However, owing to a limited understanding of phage biology during that era and the inefficient phage preparation processes, phage therapy was gradually supplanted by antibiotics, which exhibited a broad spectrum of antimicrobial activity and robust efficacy. Consequently, enthusiasm for phage research and its application in anti-infective therapy dwindled over subsequent decades. With the widespread use, and at times, misuse of antibiotics, bacterial drug resistance has become an increasingly grave concern, resulting in a near cessation of new antibiotic research and development in the 21st century. Faced with the critical problem of bacterial drug resistance, humanity may confront a “post-antibiotic era” characterized by the absence of effective antibiotics (Luepke et al., 2017). Thus, there exists an urgent necessity to discover new alternatives to antibiotics for combating infections, such as plant extracts, honey, propolis, synbiotics, antimicrobial peptides, vaccines, antibodies, pattern recognition receptors, probiotics, metals and antimicrobial enzymes (Gupta and Sharma, 2022; Roque-Borda et al., 2022; MaChado et al., 2023). Meanwhile, phage therapy has been rekindled and is gaining renewed attention and development (Rahimi-Midani et al., 2021). Although the Food and Drug Administration (FDA) has not yet authorized any phage products for clinical use, numerous studies and phase I/II clinical trials have demonstrated the safety and efficacy of phage therapy (Bao et al., 2020; Mulzer et al., 2020; Petrovic Fabijan et al., 2020; Rubalskii et al., 2020; Dedrick et al., 2021; Johri et al., 2021; Wu et al., 2021). Both the European Union and the United States initiated clinical trials of phage therapy in 2013 and 2016, respectively, which initially substantiated the efficacy and safety of phage therapy against drug-resistant bacterial infections (Jault et al., 2019; Poirel et al., 2020; Uyttebroek et al., 2022). Phage therapy is widely regarded as one of the most promising avenues for combatting pathogenic bacteria in humans, including drug-resistant *S. aureus* (Álvarez et al., 2019). This paper reviews the biological characteristics of phages, the mechanisms and advantages of phage therapy, as well as the research and application of phage therapy in the prevention and management of drug-resistant *S. aureus* infections. We also investigate the challenges of phage therapy, explore promising research directions and technological approaches, and envisage the future of phage therapy. This endeavor is aimed at offering guidance and insight for the study and implementation of phage-based treatment for drug-resistant *S. aureus* infections.

## 2 Biological features and classification of phages

Phages are viruses that infect bacteria (Clokie et al., 2011; Abedon and Murray, 2013; Isaev et al., 2021a; Isaev et al., 2021b). They consist of an outer protein shell and inner genetic material composed of nucleic acids. Phages lack complete cellular structures and exist as minute entities. Most of them can remain active in environments with a pH range of 5 to 9 and utilize bacteria, as hosts for their reproduction (Salmond and Fineran, 2015; Anand et al., 2020; Li Z. et al., 2021). Bacteriophages hold significant roles within ecosystems, influencing the structure and development of bacterial communities in their natural habitats (Simmonds et al., 2017). Due to their widespread presence and abundance, it is believed that phages are involved in approximately 20-40% of bacterial lysis events (Chevallereau et al., 2022). In natural settings, the evolution of phages is, in turn, impacted by the density and diversity of bacterial populations. The ability of phages to infect different hosts, known as their host range, is a highly adaptable trait, with the density, diversity, and quality of potential hosts being crucial factors influencing this trait (Meyer et al., 2012). At a fundamental level, expanding the host range benefits phages by allowing them to infect a wider array of hosts. However, phages with an extended host range may experience ecological costs, such as reduced replication rates in new hosts, and evolutionary costs, resulting in decreased performance in their original hosts. In contrast, phages can narrow their host range when they encounter an abundance of high-quality hosts in the microbial community (Holtzman et al., 2020; Sant et al., 2021). The ability to bind to new receptors is a pivotal step in the evolution of phage host range, often brought about by mutations in genes that encode phage tail proteins. Hosts with distinct receptors can drive the evolution of diverse phage genotypes, each with a unique host range (De Sordi et al., 2017; Cornuault et al., 2020).

Furthermore, phages exhibit significant diversity, characterized by variations in their virion structures (including tailed, non-tailed, enveloped, and filamentous phages), types of genetic material (double or single-stranded DNA or RNA), and gene content. Among these, double-stranded DNA tailed phages are the most prevalent in publicly accessible databases (Dion et al., 2020). In terms of morphology, all known phages that infect *S. aureus* belong to the order Caudovirales, which is also known as the tailed phages (Azam and Tanji, 2019). It is worth noting that in an update to phage taxonomy by the ICTV in August 2022, the phage classification was modified, and the order Caudovirales was replaced by a new class called “Caudoviricetes.” (Turner et al., 2023).

Phages can be classified into two types based on their different modes of action on host bacteria (Sharp, 2001; Kutter and Sulakvelidze, 2004). The first type is the virulent phage or lytic phage, which replicates and proliferates inside the host bacterial cell. Eventually, it lyses the bacteria, releasing progeny phages (Dion et al., 2020). The virulent phage attaches to specific receptors on the bacterial surface and injects its genetic material into the host for replication. The resulting progeny phages cause bacterial lysis through holin and endolysins, leading to the termination of the infection. These progeny phages are then released into the surrounding environment to initiate the killing process once again. Under normal circumstances, the second type of phage does not produce progeny phages or cause bacterial lysis. Instead, it integrates its genome into the host bacterial chromosome and transfers it to the progeny bacterium’s genome as the bacterium replicates and divides. This type of phage is referred to as a temperate phage or a lysogenic phage (Rohwer and Segall, 2015; Hampton et al., 2020). In the lysogenic cycle, phage genome (known as a prophage) replicates with host DNA, either integrated into the host chromosome or in a free plasmid-like state, forming a long-term stable coexistence with the host (Zhang et al., 2022). Subsequently, when the bacterium replicates and divides, the phage genome is transferred to the genome of the progeny bacteria, which is accompanied by vertical genetic transfer (Zhang et al., 2022). To date, phage lambda, is probably the most thoroughly studied and widely used temperate phage (Cortes et al., 2021). Phage lambda can propagate for many generations in the lysogenic cycle. The lytic-driving gene persists in prophage, but it is inhibited. Under some stressors (such as antibiotics), this lysogenic state will be induced and transformed into the lytic, which will lead to the lysis of the host (Zhou et al., 2023). Furthermore, prophage induction promotes horizontal gene transfer between bacteria and phages (Clokie et al., 2011). Prophages entering the lysis cycle will reprogram the host’s metabolism, which will be beneficial to phage replication, affect the bacterial community structure through phage-mediated host death, promote horizontal gene transfer and promote biogeochemical cycle (Zhang et al., 2022).

## 3 Mechanism of phage action and advantages of phage therapy

**Figure 1:**
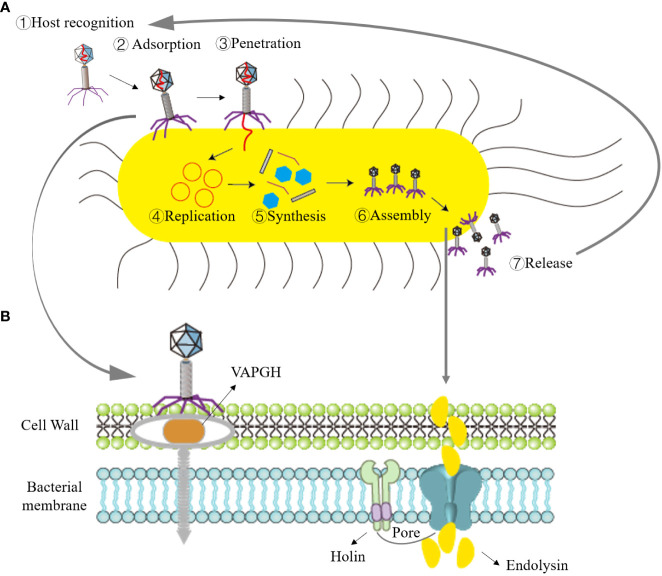
Illustration of the bacteriophage lifecycle and mechanism of bacterial lysis. **(A)** Lytic cycle, ①Recognition of host by phage; ②Absorption of bacteriophage to bacteria; ③Phage penetrates nucleic acid; ④Replication of phage nucleic acid; ⑤Synthesis of phage elements; ⑥Assembly of progeny phages; ⑦Bacterial lysis and release of progeny phages. **(B)** Role of phage endolysin in bacterial lysis: in the early stage of phage infection, the phage creates holes in the bacterial cell wall with the assistance of the VAPGH protein (which degrades a part of peptidoglycan) and injects nucleic acid into the host bacteria. Phage endolysins and holin are synthesized during the late stage of progeny phage reproduction. Holin forms pores in the bacterial inner membrane, allowing endolysins to reach the peptidoglycan.

Phage therapy typically employs virulent phages to lyse bacteria for the treatment of pathogenic bacterial infections. The lysis of bacteria by virulent phages can be divided into two processes based on their genome types: single-stranded genome phages encode lysogenic effector molecules capable of inhibiting bacterial peptidoglycan biosynthesis, while double-stranded DNA phages synthesize two proteins—holin and endolysins—that disrupt the intracellular membrane or cell wall of the host bacteria (White et al., 2011; Drulis-Kawa et al., 2015). When the phage lytic cycle is completed and progeny phages mature within the bacterial cell, holin forms pores in the bacterial inner membrane. Subsequently, endolysins pass through the inner membrane and act on the bacterial cell wall’s peptidoglycan, causing the peptidoglycan linkage bonds of the cell wall to break, leading to osmotic lysis of the bacteria. The growth and lysis of bacteria by virulent phages can be divided into four stages, as depicted in **Figure 1**: adsorption, penetration, biosynthesis, maturation, and release (Maciejewska et al., 2018).

(1) Adsorption: Adsorption is the process of specific binding between phage surface proteins and receptors on the surface of host bacteria. The principal surface proteins consist of phage-encoded polysaccharide depolymerases. These depolymerases are responsible for targeting the polysaccharide elements present in the bacterial cell envelope, including the bacterial capsule, the lipopolysaccharide (LPS) in Gram-negative bacteria, or the extracellular matrix found within biofilms (Roach et al., 2017). This specificity depends on the complementarity of their molecular structures. Adsorption represents the initial and critical step in infection. Phages can adsorb to bacteria with specific receptors, whether they are living or deceased. However, phage nucleic acid cannot enter deceased host bacteria. (2) Penetration: After adsorbing to the host bacteria, tailed phages dissolve a small hole in the host bacteria’s cell wall with the assistance of lysozymes located at the end of their tails. Virion-associated peptidoglycan hydrolases (VAPGH) are a class of lysozymes commonly found on the phage base plate. Their primary function is to locally degrade the peptidoglycan layer of the bacterial cell wall (Gordillo Altamirano Fernando and Barr Jeremy, 2019). Subsequently, through the contraction of the tail sheath, the nucleic acid contained in the phage’s head is injected into the bacterial cell, while the protein capsid remains outside the bacterial cell. (3) Biosynthesis: Once phage nucleic acid enters the bacterial cell, it initiates mRNA production through transcription and translates it into enzymes, regulatory proteins, and structural proteins related to biosynthesis. Simultaneously, it replicates a substantial quantity of nucleic acids for the progeny phages using its own nucleic acid as a template. (4) Maturation and Release: After the synthesis of proteins and nucleic acids of the progeny phages within the bacterial cytoplasm, they assemble into fully mature phages.

Once the quantity of progeny phages attains a specific threshold, holin creates openings in the bacterial inner membrane. Subsequently, endolysins traverse the inner membrane and target the peptidoglycan within the bacterial cell wall. This action results in the cleavage of the peptidoglycan linkage bonds within the cell wall, leading to osmotic lysis of the bacteria, thus liberating the progeny phages. These released phages can infect new host bacteria, ultimately leading to the lysis and demise of the host bacteria.

In comparison to traditional antibiotics, phage therapy offers several advantages. Firstly, there is an abundance and wide distribution of bacteriophages in nature, estimated at around 10 (Gupta and Sharma, 2022) types (Comeau et al., 2008; Khan et al., 2022), allowing for rapid and cost-effective screening, unlike the development of new antibiotics. Secondly, phages exhibit a remarkable level of specificity towards bacterial infections, selectively targeting the infecting bacteria without harming other bacterial species, unlike broad-spectrum antibiotics that can disrupt the balance of microbial flora (Langdon et al., 2016; Gordillo Altamirano Fernando and Barr Jeremy, 2019). Thirdly, phages naturally diminish upon the removal of host bacteria after eliminating the target bacteria *in vivo*, preventing *in vivo* accumulation and associated toxic and side effect (Gindin et al., 2019; Petrovic Fabijan et al., 2020). Fourthly, phages exclusively infect bacteria, do not enter human cells, and do not disturb the normal metabolism of human cells. Moreover, no mutagenic effects or genotoxicity on the human body have been observed (Uyttebroek et al., 2022). Fifthly, the production of phage resistance occurs at a rate 10 times slower than that of antibiotic resistance (Doss et al., 2017). Additionally, bacteriophages can play a synergistic role in combination with antibiotics without promoting antibiotic resistance (Nouraldin et al., 2016; Regeimbal James et al., 2016; Oechslin et al., 2017; Gu Liu et al., 2020; Nick et al., 2022). Lastly, phages can disrupt the structure of bacterial biofilms by secreting cell wall hydrolases and extracellular polysaccharide depolymerase, effectively combatting bacterial biofilm (Gordillo Altamirano Fernando and Barr Jeremy, 2019; Kortright et al., 2019; Royer et al., 2021).

## 4 Application of phage therapy against drug-resistant *S. aureus* infection

Bacteriophages have been widely employed in both human and animal research and treatment, with the NCBI database currently housing 69 preserved genomes of virulent *S. aureus* phages, including 26 *Podoviridae* phages and 43 *Myoviridae* phages (Kornienko et al., 2020). This section summarizes typical cases of phage therapy for drug-resistant *S. aureus* infection, as further detailed in **Table 1**.

**Table 1:** Application of phage against drug-resistant *Staphylococcus aureus* infection in animals and humans.

Bacterial strain	Infected object and type	Phage/Cocktail	Phage dose	Delivery vehicle	Route of administration	Antibiotic	Outcome	Reference
*S. aureus* CVCC 546(MRSA)	mice, mastitis	phage 4086–1	1 × 10^8^ PFU/mL,100 µL	——	mammary duct	ceftiofur sodium (5 mg/kg)	Compared to treatment with ceftiofur sodium,phage therapy was more effective in reducing the number of MRSA and inflammatory reaction	(Teng et al., 2022)
*S. aureus* ATCC 29740 (MSSA)	mice, mastitis	StaphLyse™ phage cocktail (SAML-4, SAML-12, SAML-150, SAML-229, SATA-8505)	10^8^PFU in 100 µL, two repeated doses at 8 h and 16 h after *S. aureus* infection	——	intramammary or intravenously	amoxicillin (75 µg/mL, approximately 150 times MIC)	The bacterial load could be significantly reduced by using phage cocktail or amoxicillin alone	(Brouillette et al., 2023)
multidrug-resistant *S. aureus* SA63–2498	mice, diabetic foot ulcer	AB-SA01 phage cocktail (J-Sa36, Sa83, and Sa87)	70 µL AB-SA01, equivalent to 7.9 log_10_ PFU	——	Topical wounds were coverd with AB-SA01 soaked Gauze patches and Opsite	vancomycin (150 mg/kg intraperitoneal twice daily for five consecutive days)	AB-SA01 treatment decreased the bacterial load with efficacy similar or superior to vancomycin treatment	(Kifelew et al., 2020)
*S. aureus* clinical isolate SA003	mice, mastitis	phage ΦSA012	5 × 10^9^ pfu/head for MOI of 100 or 5 × 10^7^ pfu/head for MOI of 1	——	intraperitoneally (i.p.) or intravenously (i.v.)	——	ΦSA012 treatment improved the survival rate of mice and the effect of intraperitoneal injection was better.	(Fujiki et al., 2022)
*S. aureus* ATCC 43300 (MRSA)	mice, diabetic skin wound infection	phage cocktail (MR-5 and MR-10)	10^9^ PFU/50 µL MR-5 and 10^9^ PFU/50 µL MR-10	liposome	Local administration	Two I/P doses of clarithromycin (10 mg/kg) given in 12 h intervals	Animals receiving liposomes entrapped cocktail of phages showed higher reduction in bacterial burden on all days as compared to free cocktail of phages and clarithromycin treated mice. Furthermore, entrapment of liposomes led to a significant increase in phage titer at the wound site	(Chhibber et al., 2018)
*S. aureus* JAR060131RifR (rifampicin resistance)	rabbit, fracture-related infection	phage ISP	800 µL,10^9^ PFU/mL	emulsion-based hydrogel	subcutaneous access tube injection	nafcillin subcutaneously (40 mg/kg four times per day) and rifampicin orally (40 mg/kg two times per day) for 7 days	No significant differences in bacterial load between treatment study groups, but no phage neutralization at euthanasia in the phage-loaded hydrogel group compared with that five out of eight animals receiving phage in saline developed neutralizing antibodies.	(Onsea et al., 2021)
*S. aureus* JE-2 (MRSA)	mice, lung infections	phage cocktail (phage K, phage 110, phage 134, phage 135 and phage 136)	1 × 10^8^ PFU/mouse	porous microparticles engineered from poly (lactic-co-glycolic acid (PLGA)	endotracheal delivery	——	phage-loaded microparticles showed robust mitigation of MRSA in an acute lung infection mouse model and not any gross toxicity towards human lung epithelial cells	(Kalelkar et al., 2022)
*S. aureus* clinical isolate (MSSA)	patients, prosthetic knee infection	phage cocktail (PP1493, PP1815, and PP1957)	1 mL of 1 × 10^10^ PFU/mL for each phage and final dilution 1 × 10^9^ PFU/mL	——	direct administration into the joint	Cefalexin or doxycycline as suppressive therapy	Beneficial with a clinically substantial improvement of the function for all patients	(Ferry et al., 2020)
*S. aureus* clinical isolate (MSSA)	patient, prosthetic knee infection	phage SaGR51ø1	one-time dose of 10 mL of phage (2.89 × 10^10^ PFU/mL) and daily infusions every 12 h for 6 weeks	——	intra-articular infusion	concomitant intravenously cefazolin (2 g every 8 h) for 6 weeks	negative bacterial culture and no adverse events related to bacteriophage therapy	(Ramirez-Sanchez et al., 2021)
*S. aureus* clinical isolate (MRSA)	patient, knee and hip prosthetic joint infection	phage SaWIQ0488ø1	one-time dose of 10 mL of phage (1.2 × 10^9^ PFU/mL) and daily infusions every 24 h for 3 days	——	intra-articular or intravenous infusion	Daptomycin was used for three weeks	All intraoperative bacteriological cultures were negative and there has been no evidence of recurrence	(Schoeffel et al., 2022)
*S. aureus* clinical isolate (MRSA and MSSA)	patients, bacteraemia	AB-SA01 phage cocktail (J-Sa36, Sa83, and Sa87)	10^9^ PFU per 1 mL ampoule in 50–100 mL 0.9%Il twice daily for 14 days	——	intravenously	flucloxacil-lin, cefazolin or vancomycin as antibiotic therapy supplemented by ciprofloxacin and/or rifampicin	No adverse reactions were reported, AB-SA01 intravenously administered was safe in severe drug-resistant *S. aureus* infections and it was possible that there was a synergistic effect between phages and antibiotics	(Petrovic Fabijan et al., 2020)
*Staphylococcus* spp, etc clinical isolate	patients, urinary tract infections	Pyophage cocktail	20 mL in a double-blind manner twice daily for 7 days	——	intravesical titration	either ceftriaxone (1 g once daily intravenously), amoxicillin and clavulanic acid (1 g twice daily orally), or ciprofloxacin (500 mg twice daily orally)	non-inferiority of bacteriophages in terms of efficacy compared with antibiotics, and high tolerability and safety. However, superiority of bacteriophages over placebo was not observed.	(Leitner et al., 2021)

MSSA, Methicillin-sensitive Staphylococcus aureus; MRSA, Methicillin-resistant Staphylococcus aureus; PFU, Plaque-forming unit; MOI, Multiplicity of infection.

### 4.1 *In vitro* experiments of phage therapy against drug-resistant *S. aureus* infection

Chan and Abedon initially proposed that treatment strategies could be categorized based on the number of phage types employed: “monophage therapy” using a single phage type, and “polyphage therapy” involving multiple phage types (Chan and Abedon, 2012). Titze et al (Titze and Krömker, 2020). utilized a phage mixture (7.4 × 10^9^ PFU/mL) against *S. aureus* isolate 7142 with *nuc* gene-positive, resulting in a reduction of bacterial colonies by 4.2 log_10_ CFU/mL after 12 h. Liu et al. (2022) isolated four distinct bacteriophages against drug-resistant *S. aureus* from various sources, namely nasal swabs, soil, and sheep feces: APTC-SA-2, APTC-SA-4, APTC-SA-12, and APTC-SA-13. Genome sequencing revealed that none of these bacteriophages contained virulence or antibiotic resistance genes. Sensitivity testing demonstrated that a single phage was effective against 80%-95% of *S. aureus* isolates, including 32 clinical MSSA, 17 MRSA isolates, and 2 ATCC strains. In contrast, the phage cocktail APTC-C-SA01, comprising four phages, exhibited sensitivity to over 98% of *S. aureus* isolates, displaying a strong complementary effect. Moreover, the frequency of phage-resistant mutants decreased from 0.12~0.21 × 10^–7^ to 0.9 × 10^–9^.

Studies have revealed that phages can reduce the minimal inhibitory concentration (MIC) of certain antibiotics and can act synergistically with antibiotics (Grygorcewicz et al., 2020; Gu Liu et al., 2020; Kebriaei et al., 2020a). The term phage-antibiotic synergy (PAS) was initially introduced by Comeau et al. (2007) to describe an accidental discovery where sublethal concentrations of antibiotics substantially increased the production of lytic phages by bacteria. This phenomenon was linked to the augmented biomass and biosynthetic capabilities of bacteria in the presence of antibiotic levels that were sufficient to hinder cell division without causing cell death. For the viruses, this led to a shorter latent period and a larger burst size, enabling quicker propagation and a decrease in the bacterial population. From an evolutionary perspective, the imposition of two distinct selective pressures on bacteria may reduce the likelihood of them developing potential resistance (Torres-Barceló and Hochberg, 2016). Furthermore, Gu Liu et al. (2020) revealed that phages can reduce the MIC required for bacterial strains that are already resistant to antibiotics. This effect depends on the type of antibiotic and the specific balance between the two agents, and it is significantly influenced by the host’s microenvironment. Prior studies have demonstrated the efficacy of combined phage-antibiotic therapy against *S. aureus* infections *in vitro*. For instance, Kebriaei et al. (2022) combined phage Sb-1 with daptomycin, resulting in a 2 log_10_ CFU/mL reduction in MRSA biofilm colony counts compared to the use of a single antibiotic. Similarly, Simon et al. (2021) demonstrated synergistic effects between phage Sb-1 and oxacillin. Phage Sb-1 alone reduced MRSA levels by 35%, whereas in combination with oxacillin, it reduced MRSA levels by approximately 90%. Likewise, Joo et al. (2023) confirmed that the combined application of vancomycin and bacteriophage K, compared with vancomycin or bacteriophage K alone, reduced MRSA in biofilm to less than 2 log_10_ CFU/mL, indicating significant synergy.

### 4.2 Animal experiments of phage therapy against drug-resistant *S. aureus* infection

In a study conducted by Teng Fei et al. (Teng et al., 2022) using a mouse model, a phage treatment group (with a concentration of 1 × 10^8^ PFU/mL) and a ceftiofur sodium treatment group (with a dosage of 5 mg/kg) were compared. The results showed that the phage therapy group reduced the number of drug-resistant *S. aureus* in the mammary glands by 8 log_10_ CFU/g, while the antibiotic group reduced it by 4 log_10_ CFU/g. Notably, the phage therapy group exhibited a more effective reduction in drug-resistant *S. aureus* compared to the antibiotic group. Additionally, the concentrations of tumor necrosis factor (TNF-α) and interleukin-6 (IL-6) in the treatment groups decreased significantly, especially in the phage treatment group. This indicates that phage treatment can more effectively reduce the inflammatory response in mouse mammary glands compared to ceftiofur sodium treatment. Similarly, Brouillette et al. (2023) formulated a phage cocktail called StaphLyse™, comprising five *Myoviridae* virulent *S. aureus* phages (SAML-4, SAML-12, SAML-150, SAML-229, and SATA-8505), which meet FDA safety standards for the treatment of mastitis in mice. StaphLyse™, at a concentration of 10^9^ PFU/mL, successfully lysed 709 strains of *S. aureus*, including MSSA, MRSA, and vancomycin-intermediate *Staphylococcus aureus* (VISA) strains, with a lysis rate of 100%. In *in vitro* experiments, StaphLyse™ (at approximately 10^10^ PFU/mL) reduced colony counts of *S. aureus* ATCC 29740 by 5.8 log_10_ CFU/mL after 1 h. In a mouse infection model, researchers observed that administering StaphLyse™ intramammary (10^8^ PFU in 100 µL) at 8 and 16 h after infection was the most effective treatment regimen. This reduced the bacterial load per gram of mammary tissue by 2.82 log_10_ CFU. In comparison, the amoxicillin control group (75 µg per gland, approximately 150 times the MIC of amoxicillin for *S. aureus* ATCC 29740) reduced the bacterial load per gram of mammary tissue by 4.45 log_10_ CFU. Additionally, prophylactic use of StaphLyse™ (administered 4 h prior to infection) was also effective, reducing *S. aureus* levels by 4.03 log_10_ CFU per gram of mammary gland. In another study, Kifelew et al. (2020) employed the phage cocktail AB-SA01 to treat multidrug-resistant *S. aureus* wound infections in diabetic mice. This cocktail was produced according to current good manufacturing practice (CGMP) standards and had undergone two phase I clinical trials (Lehman et al., 2019; Petrovic Fabijan et al., 2020). Results indicated that on the fourth day of treatment, the bacterial load in the phage treatment group and vancomycin treatment group had decreased by approximately 3.3 log_10_ CFU/swab compared to the normal saline control group. On the third day after treatment, compared to the normal saline control group, the bacterial load in the phage treatment group and vancomycin treatment group had decreased by approximately 8 log_10_ CFU/swab and 3.3 log_10_ CFU/swab, respectively. Additionally, Laboratory et al. found that combining AB-SA01 with vancomycin exhibited a synergistic effect, enhancing the elimination of MRSA and preventing the development of phage resistance (Kebriaei et al., 2020b).

Different routes of administration also impact the therapeutic effectiveness of phages. In a mouse model infected with SA003, Fujiki et al. (2022) administered phage ΦSA012 (GenBank database accession number NC_023573.1) intraperitoneally and intravenously. This improved the survival rate of mice from 20% to 75% and 40%, respectively. Notably, ΦSA012 exhibited a broad-spectrum host range similar to *Staphylococcus* phage K, forming plaques in 94.4% (33/35) of animal-associated MRSA and MSSA strains and 60.0% (24/40) of human-associated MRSA strains. Furthermore, pharmacokinetic studies revealed that phage ΦSA012 was detected in the liver, lung, and intestine of mice without inducing any inflammatory reactions or potential side effects in the organs of mice.

However, as an active preparation, bacteriophages exhibit low stability and long-term effectiveness at the infection site (Guerin and Hill, 2020; Bichet et al., 2021), which somewhat limits their efficacy against infections. One strategy to protect bacteriophages from the influence of the adaptive immune system is encapsulating them in a carrier (Gembara and Dąbrowska, 2021; Wdowiak et al., 2022). Chhibber et al. (2018) employed liposomes as phage delivery vehicles to encapsulate a *Myoviridae* virulent *S. aureus* phage cocktail for treating MRSA-induced skin wound infections in diabetic mouse models. The results showed that 70% of mice in the blank control group died within 24 to 48 h after MRSA infection, and the bacterial loads at the wound increased to 9 log_10_ CFU/mL. In contrast, no deaths occurred in the phage cocktail group (administered 100 µL at 10^9^ PFU/mL), clarithromycin group (administered at 10 mg/kg), or liposome-encapsulated phage cocktail group (administered 100 µL at 10^9^ PFU/mL). Over time, the bacterial loads in the treatment groups decreased, particularly in the liposome-encapsulated phage cocktail group. The bacterial loads decreased from 8.39 ± 0.72 log_10_ CFU/mL to 1.84 ± 0.17 log_10_ CFU/mL on the 7th day. The stability study of phages revealed that on the first day after administration, the phage cocktail titers in the wound of mice in the free phage cocktail treatment group and the liposome-encapsulated phage cocktail group decreased to approximately 10^5^ PFU/mL and 10^7^ PFU/mL, respectively. This indicates that liposome encapsulation can effectively prevent the decrease in bacteriophage activity and initial titer in the wound. It increases the time the bacteriophage functions at the infection site, thereby accelerating wound healing in mice.

Similarly, Onsea et al. (2021) treated rifampicin resistant *S. aureus*-induced fracture-related infections in a rabbit model by loading phages onto hydrogel. Initially, all animals in the treatment groups (Groups 3–5) received subcutaneous nafcillin (40 mg/kg, four times daily) and oral rifampicin (40 mg/kg, twice daily) for 7 days post-infection. Subsequently, Group 3 received phage injections (1 mL, 10^8^ PFU/mL in normal saline, twice daily), Group 4 underwent a single topical application of phage-loaded hydrogel (800 µL, 10^9^ PFU/mL), while Group 5 maintained the original antibiotic treatment. Results showed that infection was eradicated in 2 animals in Group 3, 1 animal in Group 4, and all animals in Group 5. Bacterial loads in the treatment groups decreased from 2 × 10^6^ CFU to approximately 10^5^ CFU. However, there was no statistically significant difference in bacterial loads between the treatment groups. Notably, the production of phage-neutralizing antibodies significantly impacts phage efficacy (Rotman et al., 2020). In this study, phage-neutralizing antibodies were only detected in the plasma of rabbits from Group 3, not in Group 4 or Group 5, suggesting that the hydrogel vehicle effectively reduces phage-neutralizing antibody production.

Additionally, Kalelkar et al. (2022) developed porous particles from degradable poly(lactic-co-glycolic acid) (PLGA) and successfully delivered a phage cocktail to the lungs of mice, effectively reducing MRSA-induced lung infections. Results indicated that compared to the control group of mice infected with MRSA, the bacterial loads in the porous particle vehicle group remained largely unchanged, indicating the vehicle had no antibacterial effect. The free phage cocktail group exhibited a reduction of approximately 1 log_10_ CFU/mg, while the porous particle group loaded with the phage cocktail demonstrated the most significant effect, with a bacterial load reduction of approximately 2 log_10_ CFU/mg. This suggests that PLGA vector synergistically enhanced the phage’s efficacy. Furthermore, the porous particle group loaded with the phage cocktail exhibited no significant toxicity to human lung epithelial cells, and the bacteria recovered from mice remained sensitive to the phage, with no emergence of phage antibodies. These findings underscore that the use of appropriate vehicles can enhance phage therapy’s effectiveness, reduce its biotoxicity, and mitigate drug resistance concerns.

### 4.3 Clinical cases/trials of phage therapy against drug-resistant *S. aureus* infection

In addition to laboratory investigations, several clinical cases have showed the promising potential of phage therapy against drug-resistant *S. aureus* infections. Ferry et al. (2020) notably improved the clinical condition of three patients with recurrent *S. aureus* clinical isolate (MSSA) prosthetic knee infections (PKIs) by employing a phage cocktail in conjunction with inhibitory antibiotics. Initially, all patients underwent “Debridement Antibiotics and Implant Retention” (DAIR) treatment and received a course of antibiotics, but these interventions proved ineffective, and the patients’ prosthetic knee joints remained severely infected. Consequently, hospital pharmacists formulated three phage cocktail preparations (PP1493, PP1815, and PP1957) at a final concentration of 10^9^ PFU/mL, which were subsequently injected directly into the joints post-DAIR surgery and joint closure. This treatment was followed by combined antibiotic therapy for 6 to 12 weeks, along with suppressive antibiotic treatment (SAT). After follow-ups at 7, 11, and 30 months, two patients exhibited only mild synovial fluid discharge and limited synovial inflammation, with no persistent or recurrent bacterial infections. Moreover, their C-reactive protein levels were negative, and clinical symptoms, such as pain-free ambulation, significantly improved. Similarly, in another clinical case involving *S. aureus* PKI infection, Ramirez-Sanchez et al. (2021) successfully cured a 61-year-old female patient using sequential intra-articular infusions of phage cocktail AB-SA01 and a single lytic phage, SaGR51ø1. Initially, after several courses of intravenous cefazolin injections, oral rifampicin and amoxicillin administration, the patient’s knee joint infection persisted. Phage therapy was then attempted, beginning with intravenous infusions of phage cocktail AB-SA01 (once every 12 h for 2 weeks) and cefazolin (once every 8 h for 6 weeks). During this treatment, bacterial cultures from the patient’s blood and synovial fluid returned negative results, and her pain subsided. However, the unavailability of further phage cocktail AB-SA01 products led to the cessation of phage therapy after two weeks, resulting in knee pain recurrence and positive *S. aureus* clinical isolate (MSSA) cultures five days later. Subsequently, during the second treatment cycle, 10 mL of phage solution SaGR51ø1 (2.89 × 10^10^ PFU/mL) was aseptically injected into the joint cavity (once every 12 h for 6 weeks), accompanied by intravenous cefazolin administration (once every 8 h for 6 weeks). After completing both treatment cycles, the patient’s synovial fluid exhibited no inflammation, bacterial cultures returned negative results, and no adverse events associated with phage therapy were reported. These findings suggest that virulent phage SaGR51ø1 exhibits a favorable safety and efficacy profile against *S. aureus* PKI infections. Furthermore, phage therapy has demonstrated efficacy and safety against PKI infections caused by MRSA (B. et al., 2022; Schoeffel et al., 2022), as seen in similar clinical cases.

As mentioned earlier, an increasing number of prominent and extensively documented clinical case reports, along with the improved accessibility of phage identification and production technologies, have contributed to the expanded adoption of phages in clinical medicine during recent years (Strathdee et al., 2023). In a single-arm, non-comparative clinical trial, an Australian hospital assessed the safety of *S. aureus* phage in humans by administering phage cocktail AB-SA01 as adjuvant therapy to 13 critically ill patients with *S. aureus* bacteremia (Petrovic Fabijan et al., 2020). All patients initially received antibiotics such as flucloxacillin (n = 10), cefazolin (n = 2), or vancomycin (n = 1), supplemented with ciprofloxacin and/or rifampicin, followed by intravenous administration of phage cocktail AB-SA01 between 4 and 10 days after antibiotic treatment initiation. Results revealed that after 90 days of phage therapy, seven patients (54%) displayed clinical improvement and survived, while six patients (46%) succumbed, including three within one week. During AB-SA01 infusion and the 4-h period thereafter, no adverse reactions such as fever, rash, or hypotension were observed. Moreover, no serious inflammatory reactions were reported in patients, signifying the safety and tolerability of AB-SA01 via intravenous administration against *S. aureus* clinical isolate (MRSA and MSSA). However, further controlled trials are needed to determine AB-SA01’s efficacy. In addition, Ooi et al. (2019) demonstrated the safety and tolerability of multiple intranasal administrations of AB-SA01 in patients with recalcitrant chronic rhinosinusitis caused by *S. aureus* clinical isolate. This study is a phase I clinical trial involving multiple ascending doses, conducted in humans for the first time. It is a single-center, prospective, and open-label trial. The trial has been registered at http://anzctr.org.au, with the identifier ACTRN12616000002482. Nine patients have been enrolled in the trial, and they are divided into three cohorts, with each cohort consisting of three patients. Each cohort received intranasal irrigation with AB-SA01 twice daily, following an ascending dose scheme: 3 × 10^8^ PFU for 7 days (cohort 1), 3 × 10^8^ PFU for 14 days (cohort 2), and 3 × 10^9^ PFU for 14 days (cohort 3). Results indicated that all participants’ vital signs, including body temperature and blood pressure, as well as clinical biochemical parameters such as hemoglobin and platelet levels, remained within normal ranges before administration, 0.5 h and 2.0 h post-AB-SA01 administration, and at the exit visit. No serious adverse events were reported, underscoring the safety and tolerability of AB-SA01. Furthermore, *S. aureus* infection diminished in all patients, with eradication achieved in two patients, suggesting promising preliminary efficacy results. However, due to the small sample size, further randomized clinical trials are necessary to confirm AB-SA01’s efficacy.

Subsequently, Leitner et al. (2021) conducted a randomized, placebo-controlled, double-blind clinical trial (Identifier, NCT03140085) to treat urinary tract infections (UTIs) in patients undergoing transurethral resection of the prostate (TURP) using intravesical phage cocktail Pyophage. Among 474 screened patients, 113 with positive urine *S. aureus* cultures entered the study (including *Staphylococcus* spp., *Streptococcus* spp., *Enterococcus* spp., and *Escherichia coli*, with colony counts ≥10^4^ CFU/mL). Patients were randomly assigned to receive treatment, resulting in 97 patients receiving one of three interventions: the phage group (n = 28), the placebo group (n = 32), or the antibiotic group (n = 37). Post-treatment assessments revealed no statistically significant differences in success rates or adverse event incidence among the three groups. Notably, intravesical phage therapy’s efficacy in treating *Staphylococcus*-induced UTIs was comparable to that of antibiotic treatment, but it did not outperform the placebo control group. This outcome may be attributed to bladder irrigation, which unexpectedly reduced bacterial loads in all groups. This randomized double-blind clinical trial reiterated phage therapy’s safety and tolerance while encouraging larger, high-quality clinical investigations.

### 4.4 Endolysin therapy against drug-resistant *S. aureus* infection

In 1958, Jacob et al. made the pioneering discovery that bacteriophages can encode a class of proteins exhibiting the capacity to lyse bacteria. These proteins, termed endolysins or lysins, serve a crucial role in the bacteriophage infection process by lysing the host bacteria. Lysins are cell wall hydrolases synthesized by phage genes during the late stages of dsDNA phage infection in bacteria. In the bacteriophage lysis replication cycle, lysins pass through cell membrane pores formed by holin and subsequently target the peptidoglycan within the bacterial cell wall. They cleave and hydrolyze vital chemical bonds in the peptidoglycan, ultimately leading to bacterial lysis and demise. This process facilitates the release of progeny phages into the extracellular environment (Abdelrahman et al., 2021).

While complete phages remain a viable antibacterial option, their limited antibacterial spectrum, intricate preclinical and clinical evaluations, and inadequate regulatory frameworks have impeded the widespread adoption of phage therapy (Abdelkader et al., 2019; Rahman et al., 2021). In contrast, lysin development has progressed more rapidly. Lysins offer advantages such as non-proliferation, high bactericidal activity, a broad host spectrum, well-defined pharmacokinetics, reduced likelihood of developing resistance, and antibodies that do not significantly diminish bactericidal activity. Consequently, lysins have emerged as significant candidates for antibiotic alternatives (Kortright et al., 2019; Röhrig et al., 2020; Abdelrahman et al., 2021; Lu et al., 2021; Murray et al., 2021; Hatfull et al., 2022; Zhu et al., 2022).

In 1959, Freimer et al. successfully purified lysins with bactericidal properties. In 2001, phage lysins were first employed as local antibacterial agents. The presence of the outer membrane in Gram-negative bacteria poses a physical barrier to lysins, impacting their effectiveness. In contrast, lysins can directly target peptidoglycan bonds and lyse the cell walls of Gram-positive bacteria, making lysins particularly effective against infections caused by Gram-positive bacteria (Abdelrahman et al., 2021; Murray et al., 2021). As a result, extensive research has focused on combating Gram-positive bacteria, especially drug-resistant *S. aureus*, with lysins. For example, lysin LysP108 exhibited up to 90% antimicrobial activity against MRSA and hindered bacterial biofilm formation (Lu et al., 2021). The catalytic domain of lysin CHAP LysGH15 reduced *S. aureus* levels in milk by approximately 2.5 log_10_ CFU/mL after 8 h at 4°C (Yan et al., 2021). Chimeric lysin ClyC decreased MRSA suspensions *in vitro* and in mice by 9 log_10_ CFU/mL and 2 log_10_ CFU/mL, respectively, and exhibited synergy with penicillin (Li X. et al., 2021). Recombinant lysin XZ.700 inhibited *S. aureus* clinical isolate proliferation and skin colonization (Pallesen et al., 2023).

Furthermore, lysins are susceptible to degradation by gastric acid and proteases upon entering the body, posing a considerable obstacle to lysin therapy’s application. Encapsulation technology offers an effective means of protecting lysins (Haddad Kashani et al., 2017; Gondil and Chhibber, 2021; Murray et al., 2021). Portilla et al. (2020) encapsulated LysRODI in pH-sensitive liposomes, which reduced *S. aureus* clinical isolate suspensions in weakly acidic (pH 5) environments and *S. aureus* within biofilms by 2 log_10_ CFU/mL. Yao et al. (2021) encapsulated chimeric lysin ClyC in alginate hydrogel, preserving ClyC’s stability while reducing cytotoxicity resulting from high concentrations (250 µg/mL) of ClyC in local infections. The researchers found that the cumulative release of ClyC from ClyC-alginate hydrogel in Tris buffer at 37°C after 72 h was approximately 23 ± 0.45%, and ClyC maintained its structural integrity without hydrolysis or degradation during gelation and release. ClyC-alginate hydrogel demonstrated almost zero cytotoxicity when incubated with BHK-21 cells, compared to 80% relative cytotoxicity for 250 µg/mL free ClyC after 24 h. In a mouse model of *S. aureus* T23-induced osteomyelitis, ClyC-alginate hydrogel reduced bacterial loads in the femur and surrounding tissues by 2 log_10_ CFU/mL, showing efficacy equivalent to that of free ClyC. However, this study warrants longer experimentation periods to assess its effects on bone healing and local inflammation adequately. Additionally, Kaur et al. observed increased bactericidal activity when delivering LysMR-5 via alginate-chitosan nanoparticles (Alg-Chi NPs) (Kaur et al., 2020). Results indicated that after 4 h of incubation at 37°C, the bacterial loads reduced to 10^6^ CFU/mL with blank Alg-Chi NPs (500 µg/mL), 10^4^ CFU/mL with free LysMR-5 (155 µg/mL), and 10^3^ CFU/mL with LysMR-5-loaded Alg-Chi NPs (500 µg/mL), compared to the initial bacterial loads of 10^8^ CFU/mL.

In 2013, Micreos introduced Gladskin (also known as Lysin SA.100 or Staphefeckt SA.100), the world’s first phage lysin product. It is primarily used as an adjuvant treatment for inflammatory skin diseases caused by MRSA (Totté et al., 2017). Staphefekt is currently registered as a (class 1) medical device in Europe, available in polycestol cream and over-the-counter gel forms. *In vitro* studies demonstrated that Staphefekt specifically targeted MSSA and MRSA, without affecting other commensal skin bacteria and without inducing drug resistance (Pastagia et al., 2013). In three case reports, local treatment with Staphefekt SA.100 inhibited drug-resistant *S. aureus*, effectively curing chronic recurrent *S. aureus*-related skin diseases that were previously resistant to antibiotics like clarithromycin, flucloxacillin, and fusidic acid cream during the early stages of treatment (Totté et al., 2017). While this study highlights Staphefekt’s potential as an alternative to conventional antibiotics for treating drug-resistant *S. aureus*-related skin infections, its efficacy and safety require further investigation through randomized controlled clinical trials.

Subsequently, in a multi-center, placebo-controlled, double-blinded, and randomized superiority trial (Home | ClinicalTrials.gov; Identifier, NCT02840955), 100 patients with moderate to severe atopic dermatitis received 12 weeks of Staphefekt treatment. Results indicated that short-term application of Staphefekt did not significantly impact non-infectious specific dermatitis patients. Simultaneous application of topical corticosteroids (TCS) and emollients may have masked any clinical benefits. However, the trial demonstrated that Staphefekt was safe and well tolerated (Totté J. et al., 2017; de Wit et al., 2019).

Gangagen, an Indian company, has developed an engineered lysin called P128 to combat *S. aureus* infections in the nasal cavity, especially MRSA infection. They have successfully completed a phase IIa clinical trial (ClinicalTrials.gov; Identifier: NCT01746654). Additionally, SAL200, another endolysin candidate for treating SaB, is currently undergoing a phase IIa clinical trial (Jun et al., 2017; Abdelkader et al., 2019; Indiani et al., 2019). Furthermore, Exebacase, a lysin designed for targeting MRSA (also known as CF-301 or PlySs2), has completed phase II clinical trials in humans (ClinicalTrials.gov; Identifier: NCT03163446). These trials have demonstrated the safety and efficacy of lysin and its synergistic effects when combined with antibiotics (Fowler et al., 2020). Currently, Exebacase is being investigated in experimental treatments for MRSA bacteremia and endocarditis patients in phase III clinical trials (ClinicalTrials.gov; Identifier: NCT04160468). If successful, it will become the first lysin-based drug (Traczewski Maria et al., 2021).

## 5 Future perspectives

Bacteriophages exhibit diverse characteristics and can be readily engineered. They possess the ability to selectively target bacteria without affecting eukaryotic cells, rendering them advantageous in comparison to antibiotic therapy. In the “post-antibiotic era,” they are poised to play a pivotal role in combating superbugs (Parasion et al., 2014). However, phages confront several challenges and hurdles in clinical applications (Hu and Tong, 2021): (1) Phage capsid proteins, being biological macromolecules with significant immunogenicity, can trigger the body’s immune clearance mechanisms against phage agents, consequently diminishing the effective phage dosage. (2) Prolonged antagonistic coevolution between bacteriophages and bacteria results in an incomplete level of host specificity. Moreover, phages can acquire, carry, and transmit bacterial gene fragments, posing the risk of disseminating drug-resistant genes (Ando et al., 2015). (3) Phages possess a high degree of specificity for recognizing and infecting host bacteria, which narrows their range of action and limits the broad application of phage preparations. (4) Similar to antibiotics, repetitive use of phage preparations can lead to bacterial tolerance against phage infection. (5) Although phage therapy has not been associated with serious toxicity or side effects, phages can release substantial endotoxins when lysing Gram-negative bacteria, raising safety concerns (Matsuda et al., 2005). (6) Clinical evidence supporting the efficacy of phage therapy remains insufficient. Existing randomized controlled clinical trials have not yielded robust results. Phage therapy often serves as an adjunct to combination therapy, complicating the assessment of phage’s independent therapeutic impact (Ooi et al., 2019; Petrovic Fabijan et al., 2020; Leitner et al., 2021). (7) There is a dearth of standards and guidelines for the clinical application of phages, including standardized treatment protocols and efficacy evaluation criteria (Matsuda et al., 2005; El Haddad et al., 2019; Suh Gina et al., 2022). (8) Ambiguity surrounds the ownership of intellectual property rights for phages. Given their natural origin and ease of isolation from the environment, protecting phages under existing legal frameworks is challenging, which dampens enthusiasm among phage practitioners and research and development companies (Gordillo Altamirano Fernando and Barr Jeremy, 2019). (9) Clinical practitioners, patients, and their families often lack knowledge about phages and phage therapy, leading to inadequate or erroneous implementation of phage treatment.

Although exciting progress has been made in phage therapy, more in-depth research is needed in the following aspects to improve the potential future clinical use of phage therapy in the future (Heilmann et al., 2010; Chen et al., 2018; Al-Ishaq et al., 2021). (1) Efforts to diminish the immunogenicity of phages by masking or modifying their antigenic epitopes can enhance their efficacy against systemic infections (Łobocka et al., 2021). In addition, encapsulating phages with liposomes, nanomaterials, or hyaluronic acid can also mitigate immune clearance (Sarhan and Azzazy, 2015; Singla et al., 2016; Kaur et al., 2021; Tabare et al., 2023). (2) Addressing bacterial resistance to phages is paramount. Engineered phage technology can be employed to design phage receptors within highly conserved regions of pathogenic bacteria (Ojala et al., 2013; Smith et al., 2023). Continuous refinement and optimization of phage cocktail formulations aligned with clinical needs can help counteract phage resistance (Samson et al., 2013; Barbu et al., 2016; Piel et al., 2022). (3) Genetic engineering can be utilized to modify the receptor binding proteins (RBPs) of phages, expanding their host range (Rehman et al., 2019). (4) Standardizing phage preparations for clinical treatment necessitates updates in regulatory strategies to accommodate the complexity of phage products (Cooper et al., 2016; Suh Gina et al., 2022). (5) The unique characteristics of phage self-replication and growth in clinical settings present challenges in determining optimal treatment conditions, including timing, dosage, and administration methods. Although some studies have elucidated the pharmacokinetic principles of phage self-replication and growth (Sarhan and Azzazy, 2015), considerable research is needed to guide drug utilization. This area remains a focal point for future research.

In conclusion, despite the challenges ahead, phage therapy holds great promise in the face of increasing antibiotic resistance. With proper oversight and intellectual property policies in place, phages are expected to play a more significant role in the field of life sciences. The clinical application and industrial development of phage therapy are poised for growth in the near future.

## Author contributions

KL: Methodology, Data curation, Writing – original draft, Writing – review & editing. CW: Methodology, Visualization, Writing – original draft, Writing – review & editing. XZ, XG: Methodology, Validation, Writing – review & editing. WL, YY: Data curation, Investigation, Writing – review & editing. RZ, HS: Conceptualization, Methodology, Supervision, Funding acquisition, Project administration, Writing – review & editing. All authors contributed to the article and approved the submitted version.
